# *ACTN3* R577X and *ACE* I/D gene variants influence performance in elite sprinters: a multi-cohort study

**DOI:** 10.1186/s12864-016-2462-3

**Published:** 2016-04-13

**Authors:** Ioannis D. Papadimitriou, Alejandro Lucia, Yannis P. Pitsiladis, Vladimir P. Pushkarev, Dmitry A. Dyatlov, Evgeniy F. Orekhov, Guilherme G. Artioli, João Paulo L. F. Guilherme, Antonio H. Lancha, Valentina Ginevičienė, Pawel Cieszczyk, Agnieszka Maciejewska-Karlowska, Marek Sawczuk, Carlos A. Muniesa, Anastasia Kouvatsi, Myosotis Massidda, Carla Maria Calò, Fleur Garton, Peter J. Houweling, Guan Wang, Krista Austin, Anastasiya M. Druzhevskaya, Irina V. Astratenkova, Ildus I. Ahmetov, David J. Bishop, Kathryn N. North, Nir Eynon

**Affiliations:** Institute of Sport, Exercise and Active Living (ISEAL), Victoria University, Victoria, 8001 Australia; Universidad Europea and Research Institute Hospital 12 de Octubre, Madrid, Spain; FIMS Reference Collaborating Centre of Sports Medicine for Anti-Doping Research, University of Brighton, Brighton, UK; Clinical Physiology Laboratory, Urals Research Centre for Radiation Medicine, Chelyabinsk, Russia; Ural State University of Physical Culture, Chelyabinsk, Russia; School of Physical Education and Sport, University of Sao Paulo, Sao Paulo, Brazil; Department of Human and Medical Genetics, Vilnius University, Vilnius, Lithuania; Department of Physical Culture and Health Promotion, University of Szczecin, Szczecin, Poland; Faculty of Physical Activity, Universidad Europea de Madrid, Alcobendas, Spain; Department of Genetics, Development and Molecular Biology, Aristotle University of Thessaloniki, Thessaloniki, Greece; Department of Life and Environmental Sciences, University of Cagliari, Cagliari, Italy; Sports Genetics Laboratory, St Petersburg Research Institute of Physical Culture, St. Petersburg, Russia; Department of Physiology, St Petersburg State University, St. Petersburg, Russia; Sport Technology Research Centre, Volga Region State Academy of Physical Culture, Sport and Tourism, Kazan, Russia; Murdoch Childrens Research Institute, Melbourne, Australia; Department of Sport Education, Academy of Physical Education and Sport, Gdansk, Poland

**Keywords:** *ACTN3*, *ACE*, Genomics, Athletic performance, Exercise, Athletes, Sprint, α-actinin-3

## Abstract

**Background:**

To date, studies investigating the association between *ACTN3* R577X and *ACE* I/D gene variants and elite sprint/power performance have been limited by small cohorts from mixed sport disciplines, without quantitative measures of performance. *Aim*: To examine the association between these variants and sprint time in elite athletes.

**Methods:**

We collected a total of 555 best personal 100-, 200-, and 400-m times of 346 elite sprinters in a large cohort of elite Caucasian or African origin sprinters from 10 different countries. Sprinters were genotyped for *ACTN3* R577X and *ACE* ID variants.

**Results:**

On average, male Caucasian sprinters with the *ACTN3* 577RR or the *ACE* DD genotype had faster best 200-m sprint time than their 577XX (21.19 ± 0.53 s vs. 21.86 ± 0.54 s, *p* = 0.016) and *ACE* II (21.33 ± 0.56 vs. 21.93 ± 0.67 sec, *p* = 0.004) counterparts and only one case of *ACE* II, and no cases of *ACTN3* 577XX, had a faster 200-m time than the 2012 London Olympics qualifying (vs. 12 qualified sprinters with 577RR or 577RX genotype). Caucasian sprinters with the *ACE* DD genotype had faster best 400-m sprint time than their *ACE* II counterparts (46.94 ± 1.19 s vs. 48.50 ± 1.07 s, *p* = 0.003). Using genetic models we found that the *ACTN3* 577R allele and *ACE* D allele dominant model account for 0.92 % and 1.48 % of sprint time variance, respectively.

**Conclusions:**

Despite sprint performance relying on many gene variants and environment, the % sprint time variance explained by *ACE* and *ACTN3* is substantial at the elite level and might be the difference between a world record and only making the final.

**Electronic supplementary material:**

The online version of this article (doi:10.1186/s12864-016-2462-3) contains supplementary material, which is available to authorized users.

## Background

Although the likelihood of becoming an elite sprint/power athlete is likely influenced by genetic factors [1, 2] only a handful of genes have been associated with sprint performance. Currently the most promising candidate gene is the *ACTN3,* which encodes the sarcomeric protein α-actinin-3 in skeletal muscle fibres [3]. The expression of α-actinin-3 protein is almost exclusively restricted to fast, glycolytic, type 2X fibres, which are responsible for producing ‘explosive’, powerful contraction [4]. Homozygosity for common null single nucleotide polymorphism (577XX, rs1815739) in the *ACTN3* gene results in complete deficiency of the α-actinin-3 protein in an estimated 18 % of humans worldwide [5], and the *ACTN3* RR genotype has been associated with elite sprint/power athletic performance in several independent cohorts of elite athletes [2].

A higher frequency of the *ACTN3* 577RR genotype (and lower frequency of the α-actinin-3 deficient, 577XX genotype) in elite sprint/power athletes (i.e., sprinters, jumpers, and throwers) was originally found in a case (athletes):control (non-athletes) association study with Australian subjects [3]. This finding was independently replicated in Finnish [6], Greek [7], Russian [8], Israeli [9] and Japanese [10] national/international level athletes. No Olympic-level sprinter has yet been identified with the 577XX genotype [3, 7, 9, 11]. Taken together, these association studies suggest that α-actinin-3 deficiency is detrimental to optimal fast muscle function at the extremes of sprint and power performance. In support of this, mice lacking α-actinin-3 (*Actn3* knockout mice, mimic the 577XX genotype in humans) demonstrate a shift in the physiological and metabolic properties of ‘fast’ glycolytic muscle fibres (type 2B) towards a slower, oxidative muscle phenotype (types 1 and 2A), which are responsible for postural and endurance activities [12] and *ACTN3* 577XX humans show lower proportion of fast-twitch muscle fibers [13, 14] and lower levels of testosterone [15].

Another candidate gene associated with elite performance is the *ACE* I/D. Besides regulating blood pressure, *ACE* may influence skeletal muscle function [16]. Indeed, ACE catalyses the conversion of the vasoconstrictor agent angiotensin I into, angiotensin II, which not only as a more potent vasoconstrictor but also as a muscle growth factor that is involved in overload-induced muscle hypertrophy [17]. The *ACE* D allele is usually associated with higher *ACE* activity, thereby potentially resulting in higher angiotensin II levels [16] and higher proportion of fast, glycolytic, type 2X muscle fibres [18]; as such, this allele could theoretically favour sprint/power-oriented performance. However, collectively association studies performed with elite sprint/power athletes are inconclusive [19–24].

One of the limitations of most studies above, investigating the association between the *ACTN3* R577X or the *ACE* I/D genotype and sprint/power performance, is grouping together sprint and power athletes from heterogeneous mixed sport disciplines and events (e.g., sprinters, jumpers, throwers, swimmers, and team sport athletes). This approach, while understandable given the very low number of world-class sprinters, reduces the accuracy of the phenotype. Furthermore, to date, only one report involving World-class sprinters of African ancestry [11], has studied the association between *ACTN3* R577X and *ACE* I/D polymorphism and athletic status; the lack of positive findings was attributable, at least partly, to the very low frequency of the *ACTN3* 577XX and *ACE* II genotypes in the African population, which almost eliminates the possibility of detecting an association. Here, we sought to address these limitations and provide deeper insights into the influence of the *ACTN3* R577X and the *ACE* I/D variants on sprint performance by using a quantitative collaborative approach and by studying the influence of genotype on actual sprint performance.

Therefore, the aim of the present study was to examine the association between the *ACTN3* R577X and the *ACE* I/D variants and 100-, 200-m and 400-m best personal times, in a large, performance-homogenous, cohort of elite Australian, Brazilian, Greek, Jamaican, Italian, Polish, Russian, Lithuanian, Spanish and US sprinters.

## Methods

### Participants

A total of 555 personal best 100-m, 200-m and 400-m sprint times of 346 elite pure sprinters from Australia, Brazil, Greece, Jamaica, Italy, Lithuania, Poland, Russia, Spain and US were analyzed (Tables 1 and 2). The sprinters from Australia, Greece, Poland, Lithuania and Russia were all Caucasians (189 male and 66 female) whereas a total of 91 male Brazilian, Jamaican, US, Italian and Spanish athletes were from African lineage. The sprinters’ best personal records (tail wind < 2 m/s when provided) in official competitions were found online (www.iaaf.org) or provided by coaches or the athletes themselves and independently corroborated (Tables 1 and 2). The sprinters’ best personal sprint times, grouped according to ethnic-background (Caucasians / Africans mixed linage athletes were excluded) and event (100-, 200- or 400-m), were standardized and relatively expressed to the relevant current World record. We included only ‘pure’ sprinters with the personal sprint time within 15 % of the current relevant World record of the examined event. Thus, the following world records were used to calculate the inclusion criterion: in the male sprinters of African ancestry, 9.58 s in 100-m and 19.9 s in 200-m (current World record holder: Usain Bolt), and 43.18-s in 400-m (current World record holder: Michael Johnson); in female sprinters of African ancestry, 10.49 s in 100 m and 21.34 s in 200-m (current World record holder: Florence Griffith-Joyner); in the Caucasian male sprinters, 9.99 s in 100-m (best record holder: Christophe Lemaitre), 19.72 s in 200-m (best record holder: Pietro Mennea), and 43.45 s in 400-m (best record holder: Jeremy Wariner); and in female Caucasian sprinters, 10.77-s in 100-m (best record holder: Ivet Lalova) and 21.71 s and 47.60s in 200-m and 400-m respectively (best World record holder: Marita Koch).

**Table 1:** The 100-m, 200-m and 400-m best sprint times (average±SD) in males according to *ACTN3 R577X* genotype distribution

	Caucasians				African Ancestry	
Running event/Genotype	RR	RX	XX	RR + DD + RX	RR	RX
100 m Male	10.55 ± 0.27 (*n* = 35)	10.58 ± 0.33 (*n* = 44)	10.77 ± 0.31 (*n* = 10)	10.56 ± 0.32 (*n* = 47)	10.26 ± 0.35 (*n* = 22)	10.28 ± 0.30 (*n* = 11)
200 m Male	21.19 ± 0.53 (*n* = 35)	21.29 ± 0.61 (*n* = 36)	21.86 ± 0.54* (*n* = 8)	21.17 ± 0.52 (*n* = 41)	20.53 ± 0.64 (*n* = 23)	20.98 ± 0.72 (*n* = 11)
400 m Male	46.90 ± 1.29 (*n* = 44)	47.41 ± 1.43 (*n* = 46)	47.55 ± 1.42 (*n* = 9)	46.82 ± 1.28 (*n* = 43)	46.49 ± 1.66 (*n* = 18)	47.29 ± 1.69 (*n* = 7)

*200-m (RR vs. RX vs. XX) *P* < 0.016

**Table 2:** The 100-m, 200-m and 400-m best sprint times (average±SD) in males according to *ACE I/D* genotype distribution

	Caucasians				African Ancestry		
Running event/Genotype	DD	ID	II	XX + II + ID	DD	ID	II
100 m Male	10.62 ± 0.32 (*n* = 33)	10.60 ± 0.29 (*n* = 28)	10.70 ± 0.30 (*n* = 13)	10.63 ± 0.29 (*n* = 42)	10.07 + 0.38 (*n* = 5)	10.27 + 0.32 (*n* = 13)	10.35 + 0.49 (*n* = 6)
200 m Male	21.33 ± 0.56 (*n* = 27)	21.25 ± 0.51 (*n* = 24)	21.93 ± 0.67** (*n* = 11)	21.47 ± 0.64** (*n* = 36)	20.53 + 0.70 (*n* = 9)	20.47 + 0.57 (*n* = 11)	21.16 + 0.42 (*n* = 4)
400 m Male	46.94 ± 1.19 (*n* = 35)	47.24 ± 1.40 (*n* = 43)	48.50 ± 1.07** (*n* = 11)	47.49 ± 1.44** (*n* = 58)	46.57 + 1.7 (*n* = 11)	47.08 + 1.29 (*n* = 7)	46.85 + 1.52 (*n* = 4)

**200-m (DD vs. ID vs. II) *P* < 0.004; **400-m (DD vs. ID vs. II) *P* < 0.003

**200-m (RR + DD + RX see Table 1 vs. XX + II + ID) *P* < 0.002; **400-m (RR + DD + RX see Table 1 vs. XX + II + ID) *P* < 0.001

### Genotyping

Genomic DNA was isolated from buccal epithelium, or white blood cells. The Australian, Greek, Italian, Lithuanian, US, Jamaican, Russian (from St Petersburg) and Spanish sprinters’ DNA samples were genotyped using the polymerase chain reaction (PCR)-restriction fragment length polymorphism (RFLP) method as previously described [4].

The DNA samples of the Polish, Brazilian, Russian (from Chelyabinsk), were genotyped in duplicates using an allelic discrimination assay on a Step One Real-Time PCR instrument (Applied Biosystems, Carlsbad, California, USA) with Taqman® probes. To discriminate *ACTN3* R577X (rs1815739) alleles, TaqMan® Pre-Designed SNP Genotyping Assay was used (assay ID: C_590093_1_), including appropriate primers and fluorescently labeled (FAM and VIC) MGB™ probes to detect the alleles.

Genotyping of the *ACE* I/D polymorphism, in all cohorts, was carried out as previously described [25].

### Statistical analysis

To compare the sprinters’ records between all genotypes we used the one-way analysis of variance (ANOVA). The *Tukey’s post*-*hoc test* was used to determine statistical significant difference among the genotype groups. The level of significance was set at 0.05. Using the Simple Interactive Statistical Analysis website (SISA; www.quantitativeskills.com/sisa/) genotype interactions on sprint performance were further assessed using correlation analysis as previously described [26]. Briefly, three genetic models (additive model and two dominant models assuming complete dominance of each allele) were tested. The Additive genetic model consisted of 0, 0.5 and 1, to represent R allele homozygotes, RX heterozygotes and homozygotes for the X allele, respectively; for the R allele dominant or X allele dominant genetic models, the corresponding values were 0, 0, 1 or 0, 1, 1, respectively. The proportion of the genetic contribution to phenotypic variance explained by each genetic model was estimated by expressing *r*^2^ from the correlation analyses (taken as an estimate of percentage variance explained under the model) as a percentage of the variance explained by genotype effects in the model-free ANOVAs. This proportion was compared for each model to predict the most accurate model tested.

## Results

The personal best 100-, 200- and 400-m sprint times (±SD), according to the *ACTN3* and *ACE* genotype and distribution, are presented in Tables 1 and 2.

### *ACTN3* and *ACE* genotypes influence 200-m best-personal sprint time in male athletes

**Fig. 1:**
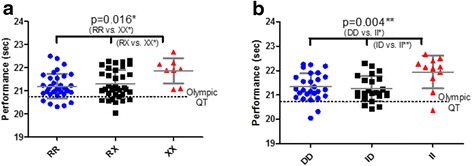
**a** Individual 200-m running times (±SD) in male Caucasian sprinters according to their *ACTN3* R577X genotype and the qualifying times (QT) for the Olympic Games (20.65 s). **b** Individual 200-m running times (±SD) in male Caucasian sprinters according to their *ACE* I/D genotype and the qualifying times (QT) for the Olympic Games (20.65 s)

In male Caucasian sprinters a significant association was detected between *ACTN3* genotype and 200 m best-personal sprint time. Using Tukey’s Multiple Comparison Test both *ACTN3* 577RR, (−0.66, 95 % CI −0.20 to −0.12) and 577RX (−0.56, 95 % CI −1.10 to −0.02) individuals were significantly faster than 577XX individuals (*P* < 0.05). We found the R allele dominant model (RR/RX vs. XX) had the best fit explaining 9.65 % of sprint time (*P* = 0.005), compared to the additive (7.28 %, *P* = 0.01) and the X allele dominant model (2.77 %, *P* > 0.05) in the correlation analysis. The percentage of the observed variance (coefficient of determination, r^2^) explained by the *ACTN3* genotype using this recessive model was 0.92 %. The *ACTN3* RR and *ACTN3* RX groups were not significantly different, indicating the presence of one or two R allele does not have a dose dependant effect on 200 m sprint speed in elite athletes (Fig. 1a). In elite male African athletes (*n* = 92), there was some evidence for a dose effect of the *ACTN3* R allele and 200 m sprint speed (Table 1). Using an unpaired *t*-test, the *ACTN3* RR individuals had (on average) a faster best-personal sprint time than *ACTN3* RX individuals (−0.45, 95 % CI, 0.95 to −0.04).

In male Caucasian sprinters a significant association was detected between the *ACE* genotype and 200 m sprint time. Using Tukey’s Multiple Comparison Test both *ACE* DD, (−0.60, 95 % CI −1.09 to −0.11) and *ACE* ID (−0.68, 95 % CI −1.18 to −0.18) individuals had significantly faster sprint time than *ACE* II individuals (*P* < 0.05). Again, coding the genotypes to test gene models found the D allele dominant model (DD/ID vs. II) had the best fit explaining 16.4 % of variance (*P* = 0.001), compared to the additive (8.0 %, *P* = 0.02) and the I allele dominant (1.2 %, *P* > 0.05) model. The percentage of observed variance (coefficient of determination, r^2^) explained by the *ACE* genotype using this recessive model was 1.48 % (Fig. 1b).

### *ACTN3* and *ACE* genotypes influence 400-m best-personal sprint time in elite male athletes

In male Caucasian sprinters a significant association was detected between the *ACE* genotype and 400-m sprint time. Using Tukey’s Multiple Comparison Test both *ACE* DD, (−1.55, 95 % CI −2.61 to −0.48) and *ACE* ID (−1.25, 95 % CI −2.29 to −0.21) individuals had significantly faster sprint time than II individuals (*P* < 0.05). Using genetic models we found, again, that the D allele dominant model (DD/ID vs. II) had the best fit explaining 11.39 % of 400 m sprint time (*P* = 0.001), compared to the additive (9.78 %, *P* = 0.002) and I allele dominant (3.97 %, *P* > 0.05). The percentage of observed variance (coefficient of determination, r^2^) explained by the *ACE* genotype using this recessive model was 1.48 %.

**Fig. 2:**
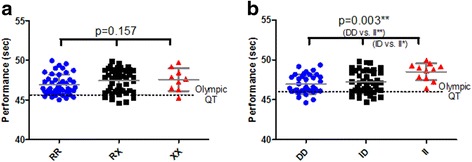
**a** Individual 400-m running times (±SD) in male Caucasian sprinters according to their *ACTN3* R577X genotype and the qualifying times (QT) for the Olympic Games (45.90s). **b** Individual 400-m running times (±SD) in male Caucasian sprinters according to their *ACE* I/D genotype and the qualifying times (QT) for the Olympic Games (45.90s)

The *ACE* DD and *ACE* ID groups were not significantly different in sprint time, indicating the presence of one or two D allele does not have a dose dependant effect on 400-m best-personal sprint time in elite athletes (Fig. 2b). No cases of *ACE* II male sprinters with a best-personal 400-m sprint time faster than the Olympics qualifying time (45.90s) were noted.

### No genotype differences were detected in 100-m sprint performance in both Caucasian and African ancestry sprinters

**Fig. 3:**
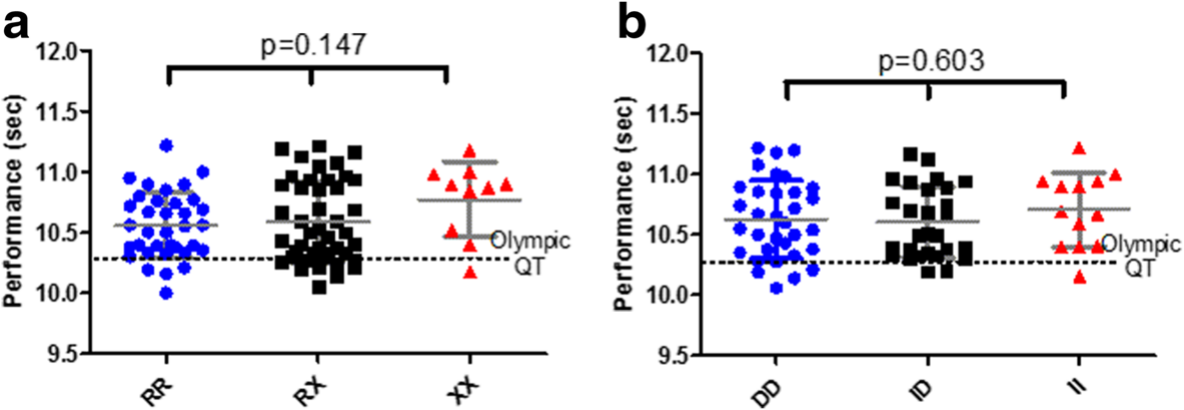
**a** Individual 100-m running times (±SD) in male Caucasian sprinters according to their *ACTN3* R577X genotype and the qualifying times (QT) for the Olympic Games (10.24 s). **b** Individual 100-m individual running times (±SD) in male Caucasian sprinters according to their *ACE* I/D genotype and the qualifying times (QT) for the Olympic Games (10.24-s)

There was no statistical significant difference in 100-m best running times across the *ACTN3* R577X and *ACE* I/D genotypes (Figs. 3a and b). Caucasian (*n* = 66) females were assessed separately and showed similar running times to males, across genotypes.

### No cumulative genotype effect on 200-m sprint performance

In male Caucasian sprinters, a significant interaction was detected between the genotype combination of *ACTN3* RR/RX and *ACE* DD with 200-m sprint speed (Tables 1 and 2). Given we have found a positive associations by independently examining the *ACTN3* RR/RX and the *ACE* DD/ID genotypes in Caucasian male 200-m sprint performance, we wondered whether the combination of these positive genotypes would have a cumulative benefit to performance. A genotype score was applied (RR/RX = 2, DD/ID = 2, XX = 0, II = 0) and plotted against 200 m performance time. An ideally predisposed sprinter would receive 4; a less ideally predisposed 2 and an adversely predisposed 0. Despite hypothesising that the 200 m sprint time would be faster with a higher genotype score, the slope was close to zero (r^2^ = 0.02). Hence, there was no evidence for additive effect of these variants on 200 m sprint speed (Additional file 1).

## Discussion

In this quantitative assessment of genotype with qualifying time in 346 elite sprinters, we have shown that it is rare for humans with the α-actinin-3 deficient (*ACTN3* 577XX) and the *ACE* II genotypes to qualify for the 200-m, and 400-m competitions, respectively, at the Olympic Games. From all male sprinters’ best personal times included in this study, there were no cases of 577XX sprinters who had faster best-personal running time than 2012 Olympic qualifying standard in 200-m (20.65 s), and no cases of *ACE* DD sprinters who had best-personal running time faster than 2012 Olympic qualifying standard in 400-m (45.90s). Collectively, these findings suggest that the *ACTN3* 577XX and the *ACE* II genotypes are detrimental for 200- and 400-m sprint performance respectively.

In the present study, we have addressed some of the limitations inherent in previous athlete case–control studies regarding the association between *ACTN3* and *ACE* genotypes and elite athletic performance. Firstly, we have studied ten cohorts of elite sprinters, including the fastest sprinters on earth. Consequently, the number of ‘pure’ elite sprinters (*n* = 346) in the present study is much larger compared to previous association studies, and demonstrates the benefits of a collaborative approach that has been recommended in the field of exercise genomics [1, 27, 28]. Secondly, previous reports have grouped together sprint and power athletes from mixed sports disciplines and events [3, 7–10, 28]. Here, we have embraced a more stringent approach and included only elite ‘pure’ sprinters whose main sporting event was the 100-, 200- or 400-m sprint event. Furthermore, we set explicit criteria (100-, 200- and 400-m best personal running times within 15 % from the World record) to ensure a high performance level for the elite sprinters we studied. Thirdly, we have analysed quantitative measure with respect to *ACTN3* and *ACE* genotypes to assess the genotype effect size in both males and females separately. Only one previous study has taken a similar approach with 100-m sprinters; a subgroup analysis of male Japanese track and field athletes indicated that those harbouring the *ACTN3* 577XX genotype ran the 100-m sprint significantly slower than their 577RX and 577RR counterparts [10]. However, this study was limited by its sample size (*n* = 28) and studied only Japanese cohort.

The analyses we performed show that the male Caucasian sprinters’ best personal times are influenced by both *ACTN3* and *ACE* genotypes in an event specific manner. While similar trends were seen in African and female athletes, we highlight that larger cohorts are urgently needed for adequate genotype-performance assessments. Our analyses on male Caucasian performances found that 200- or 400-m (but not 100-m performance) is influenced by both the *ACTN3* and the *ACE* genotypes. Moreover, we showed that at the highest level of sprinting competition (i.e., elite sprinters who had best-personal sprint time faster than European or Olympic qualifying times) there was no significant overlap of the times for each genotype, suggesting that ‘every variable counts’ for achieving world-class sprinting performance. These findings, together with the separate results from 200- and 400-m sprints support the notion that performance in the longer 200- and 400-m sprinting events is influenced by both *ACTN3* R577X and *ACE* ID polymorphisms.

These genotype association differences may be related to subtle differences in the physiological performance demands of each event. In the 100-m race the athlete is required to accelerate for most of the race before reaching their absolute maximum velocity [29]. In longer sprinting events (200- and 400-m) the acceleration phase is relatively shorter, and rather, it is the ability to maintain the maximum velocity for a longer time period that is the critical factor for winning the events [30]. Acceleration relies on reaction time, centre of gravity of the body relative to the blocks, frequency of step and step length, while maintaining absolute maximal velocity requires powerful cyclic muscle contractions and efficient utilization of the energy systems (mostly lactic and ‘alactic’ anaerobic systems) that are triggered at different phases of the race [31]. Given the genotype-performance associations at longer (200- and 400-m) distances, this suggests their influence may lie greater effect on muscle’s metabolic potential (switch from P/Cr to lactic anaerobic systems) with repeated powerful contractions.

The *Actn3* knockout (KO) mouse has provided a possible explanation for the detrimental effect of α-actinin-3 deficient (577XX genotype) on elite sprinting performance. Mechanistic studies in the *Actn3* KO mouse show that this model mimics *ACTN3 XX in* humans. The wild-type WT mice that express Actn3 (equivalent to human RR/RX genotypes) prefer the anaerobic system while *Actn3* KO mice prefer the aerobic system. Metabolically, the KO mice have significantly higher activity of oxidative enzymes, and lower activity in enzymes of the anaerobic pathway. In addition, they have enhanced glycogen accumulation due to lower glycogen phosphorylase activity has been observed [32, 33]. Their fast fibre properties shift towards a slower metabolic profile which has been linked to increases in calcineurin signalling activity [34] and altered calcium handling [35]. Overall this shift towards a slower physiological and metabolic profile would be detrimental to sprint performance in *ACTN3* 577XX humans [12, 32].

While a similar mechanistic model of the ACE genotype does not exist, a possible explanation for the metabolic effect of the *ACE* genotype on sprint velocity performance may be related to fibre-type differences. Zhang et al. [18], has showed that the *ACE* II genotype is associated with higher percentage of type I, aerobic-oriented, muscle fibres while the *ACE* DD genotype would be linked with a higher percentage of type 2X, more anaerobic-oriented muscle fibres. While, more studies are required to fully elucidate the mechanism behind the potential association between the *ACE* genotype and human elite sprint performance, it is interesting to note that both *ACTN3* and *ACE* variants may independently affect muscle fibre metabolism.

An additional exploration in the present study was testing whether sprinters with the theoretical ‘favourable’ genotypes for *ACE* (DD/ID) and *ACTN3* (RR/RX) had cumulative effects on performance speed. While we did find a significant interaction for the *ACE* and *ACTN3* genotypes and sprint time (Tables 1 and 2), we have subsequently scored these variants and plotted against 200 m sprint time, and could not detect evidence for additive effect on 200 m sprint speed. Given the limited number of athletes with the *ACTN3* XX + *ACE* II genotype (and no dose dependant allele differences in RR/RX and DD/ID), as well as performance times that are approaching the limits of human performance, testing for measurable cumulative performance benefits in these elite cohorts maybe difficult to achieve.

Despite no evidence for cumulative performance benefits for the *ACE* and *ACTN3* variants, both genes have been repeatedly shown to be altered in frequency in elite athletes compared to controls. Given these associations, the notion that elite performance is a polygenic trait by nature (e.g., a phenotypic trait produced by multiple genes working together), with a minor contribution of each variant to the unique athletic phenotype, remains supported. While probability of becoming an elite athlete is thought to increase based upon having a greater number of athletic-related alleles [36–38], our initial scoring in male Caucasians demonstrates that it may be difficult to ascertain cumulative contributions to performance.

Here, we have shown for the first time quantitatively that the *ACTN3* and *ACE* genotypes account for ~1-1.5 % in sprint speed amongst elite male athletes. This difference in sprint time is substantial at the elite level and can be the difference between a world record and only making the final 16. Despite this, the predictive value of these tests remains limited. A substantial amount of performance variation remains unaccounted for and further research into both common and rare variants is still required.

## Conclusions

In conclusion, our multi-centre study has enabled us to gain insights into the effect of *ACTN3* and *ACE* genotypes on elite sprinting performance. Both *ACTN3* R577X and *ACE* ID polymorphisms modulate specific sprint phenotypes and influence the athletic status at the extremes of human performance. While the *ACTN3* R577X polymorphism seems to be more influential to 200-m performance, the *ACE* ID polymorphism would be more involved in the longer, 400-m sprint performance. With greater knowledge of both common and rare performance variants these findings might have future applications for identifying and coaching talented 200- and 400-m sprinters.

### Ethics statement

Ethical approval was obtained from the Human Research Ethics Committees of the Children’s Hospital at Westmead (2003/086), RCH Human Research Ethics Committee (35172), The Lithuanian National Committee of Biomedical Ethics, The Ethics Committee of St. Petersburg State University, The Ethics Research Committee of the University of Sao Paulo, The Ural State University of Physical Culture Ethics Committee, The Universidad Pablo Olavide Research Committee, The Ethics committee of Pomeranian Medical University, Aristotle University of Thessaloniki Research Committee, The Ethics Committee of the University of Cagliari, and UHWI/UWI/FMS Ethics Committee. All studies were conducted in accordance with the ethical standard laid down in the 1964 Declaration of Helsinki and its later amendments and all participants have signed a consent form.

## Additional file

Additional file 1:
**No cumulative genotype effect on 200-m sprint performance.** (PNG 6 kb)
