# Supplementary figures and images for: ACTN3 R577X and ACE I/D gene variants influence performance in elite sprinters: a multi-cohort study
Source: BMC Genomics. 2016 Apr 13;17:285. doi: 10.1186/s12864-016-2462-3 (PMC4831144; doi:10.1186/s12864-016-2462-3)

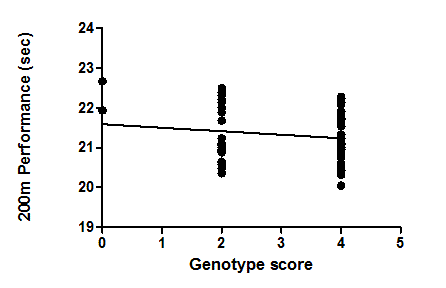

Supplement: Additional file 1: — No cumulative genotype effect on 200-m sprint performance. (PNG 6 kb) [file 12864_2016_2462_MOESM1_ESM.png]
